# New recombinant cyclohexylamine oxidase variants for deracemization of secondary amines by orthogonally assaying designed mutants with structurally diverse substrates

**DOI:** 10.1038/srep24973

**Published:** 2016-05-03

**Authors:** Guangyue Li, Peiyuan Yao, Peiqian Cong, Jie Ren, Lei Wang, Jinhui Feng, Peter C.K. Lau, Qiaqing Wu, Dunming Zhu

**Affiliations:** 1 National Engineering Laboratory for Industrial Enzymes and Tianjin Engineering Center for Biocatalytic Technology, Tianjin Institute of Industrial Biotechnology, Chinese Academy of Sciences, 32 Xi Qi Dao, Tianjin Airport Economic Area, Tianjin 300308, PR China; 2 Departments of Chemistry and Microbiology & Immunology, McGill University, Montreal, Quebec, H3A2B4, Canada

## Abstract

To further expand the substrate range of the cyclohexylamine oxidase (CHAO) from *Brevibacterium oxydans*, a library of diverse mutants was created and assayed toward a group of structurally diverse substrates. Among them, mutants T198A and M226A exhibited enhanced activity relative to wt CHAO for most (*S*)-enantiomers of primary amines and some secondary amines. While mutants T198I, L199I, L199F, M226I and M226T were more active than wt CHAO toward the primary amines, mutants T198F, L199T, Y321A, Y321T, Y321I and Y321F enhanced the enzyme activity toward the secondary amines. In particular, mutant Y321I displayed an enhanced catalytic efficiency toward 1-(4-methoxybenzyl)-1, 2, 3, 4, 5, 6, 7, 8-octahydroisoquinoline (**13**). Whereas a double mutant, Y321I/M226T, acted on (*S*)-*N*-(prop-2-yn-1-yl)-2, 3-dihydro-1H-inden-1-amine [(*S*)-**8**]. Since (*R*)-**8** is an irreversible inhibitor of monoamine oxidase and (*S*)-**13** is an intermediate of dextromethorphan, a cough suppressant drug, deracemizations of **8** and **13** were carried out with crude enzyme extracts of the respective mutants. This resulted in 51% and 78% isolated yields of (*R*)-**8** and (*S*)-**13**, respectively, each with high enantiomeric excess (93% and 99% ee). The results demonstrated the application potential of the evolved CHAO mutants in drug synthesis requiring chiral secondary amines.

Enantiomerically pure chiral amines are widely used as intermediates for agrochemicals and pharmaceuticals^1^, and they are of increasing value in organic synthesis. Recently, several kinds of biocatalysts for the synthesis of chiral amines have received significant attention. For example, transaminases^2^ and ammonia lyases^3^ are now finding applications in the industrial synthesis of chiral amines. Engineered amino acid dehydrogenases are emerging biocatalysts that are capable of using ketones as substrates for the asymmetric synthesis of chiral amines^4^. In 2002, Turner *et al.* reported a novel deracemization strategy for the preparation of optically active chiral amines^5^. This protocol was applied to the deracemization of primary^5^,^6^,^7^, secondary^8^,^9^ and tertiary amines^10^,^11^,^12^ as well as substituted pyrrolidines^13^ using recombinant monoamine oxidase (MAO-N) mutants obtained by directed evolution. The combination of biocatalytic oxidation with non-stereoselective reduction of the resulting imine by ammonia-borane generally rendered a high enantiomeric excess (ee) of a desired enantiomer from the racemate. In this context, Leisch *et al.* explored the possibility of a bacterial cyclohexylamine oxidase (CHAO), derived from *Brevibacterium oxydans* IH-35A, as an alternative biocatalyst for the synthesis of chiral primary amines using either kinetic resolution or deracemization protocol^14^. The wild-type CHAO (wt CHAO) exhibited low or no activity toward secondary and tertiary amines. We carried out site-specific mutagenesis^15^, and iterative saturation mutagenesis coupled with functional screening, to afford mutant CHAOs^16^, which increased the activity toward primary amines^15^ and extended the substrate specificity to include some secondary amines^16^. Notably, the substrate 2-methyl-1, 2, 3, 4-tetrahydroquinoline (**10**) was deracemized by a CHAO triple mutant (T198F/L199S/M226F) leading to the production of (*R*)-2-methyl-1, 2, 3, 4-tetrahydroquinoline (THQ) with 76% yield and 98% ee. This *R*-enantiomer is a valuable building block for the synthesis of a variety of THQ derivatives of pharmaceutical or clinical importance^17^,^18^,^19^,^20^,^21^,^22^.

Recently, Vergne-Vaxelaire *et al.* reported a high throughput orthogonal screening of putative nitrilases against 25 structurally diverse substrates that resulted in over one hundred new nitrilases from a genomic library of prokaryotic origin^23^. This prompted us to adopt a similar strategy to further explore the substrate specificity of CHAO. Accordingly, a library of site-directed mutants was constructed, and assayed against 18 structurally diverse amines. Among the new mutants, Y321I/M226T and Y321I were applied to deracemization of *N*-(prop-2-yn-1-yl)-2, 3-dihydro-1H-inden-1-amine (**8**) and 1-(4-methoxybenzyl)-1, 2, 3, 4, 5, 6, 7, 8-octahydroisoquinoline (**13**), respectively, as a proof of concept for the synthetic potential of the biocatalysts.

## Results

### Choice of mutation residues and construction of the mutant library

**Figure 1:**
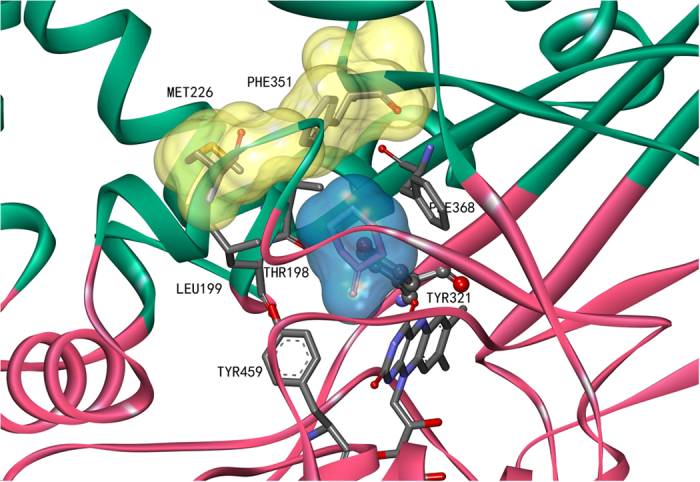
The active site of cyclohexylamine oxidase (CHAO). The substrate-binding cavity is shown in blue and the secondary cavity in yellow. The structure according to reference 24 was visualized with Accelrys Discovery Studio 4.1 (Accelrys Inc.).

The solved two-domain structures of CHAO consisting of a cofactor-binding domain and a substrate-binding domain provided a framework for the choice of amino acid residues for mutagenesis^24^. In the substrate-binding domain, the side chains of Y321, F368 and Y459 form a cage-like structure in which the amine substrate is bound (Fig. 1). This cage-like structure in CHAO is similar to that of the human monoamine oxidase B (MAO B) formed by the side chains of Y398 and Y435 which serve to orient the amine substrates and promote catalysis^25^. In addition to the substrate-binding cavity, CHAO has a second cavity that is located closer to the protein surface, and possibly serves as a substrate entrance cavity. These two cavities are separated by the side chains of amino acid residues, T198, L199, M226 and F351(Fig. 1)^24^. Hence, access to the active site from the entrance cavity is blocked by these amino acids and they are postulated to play a “gate-keeping” role in CHAO^24^.

**Figure 2:**
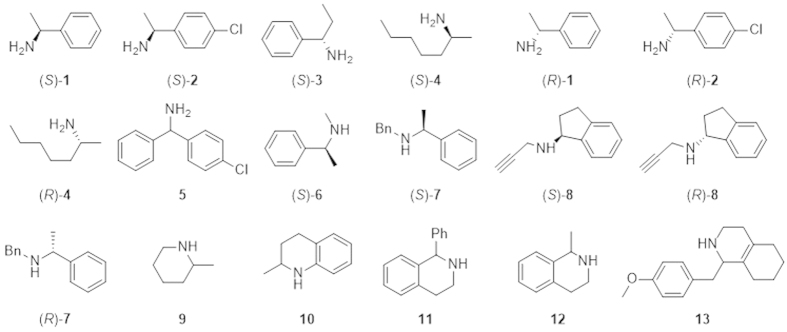
Substrates used for the activity assay.

Accordingly, residues T198, L199, M226, Y321, F351, F368 and Y459 were postulated to affect substrate entrance and binding in the catalytic cavity, thus determine the substrate specificity of CHAO. Therefore, these seven amino acids were selected as mutation targets. We adopted the strategy of Verge-Vaxelaire *et al.* in which the selected residues were mutated to amino acids of different properties to create a library of 51 designed mutants (supplementary Table S1). The mutants were assayed against a group of substrates consisting of 18 structurally diverse amines (Fig. 2).

### Activity assay of the mutant library

**Figure 3:**
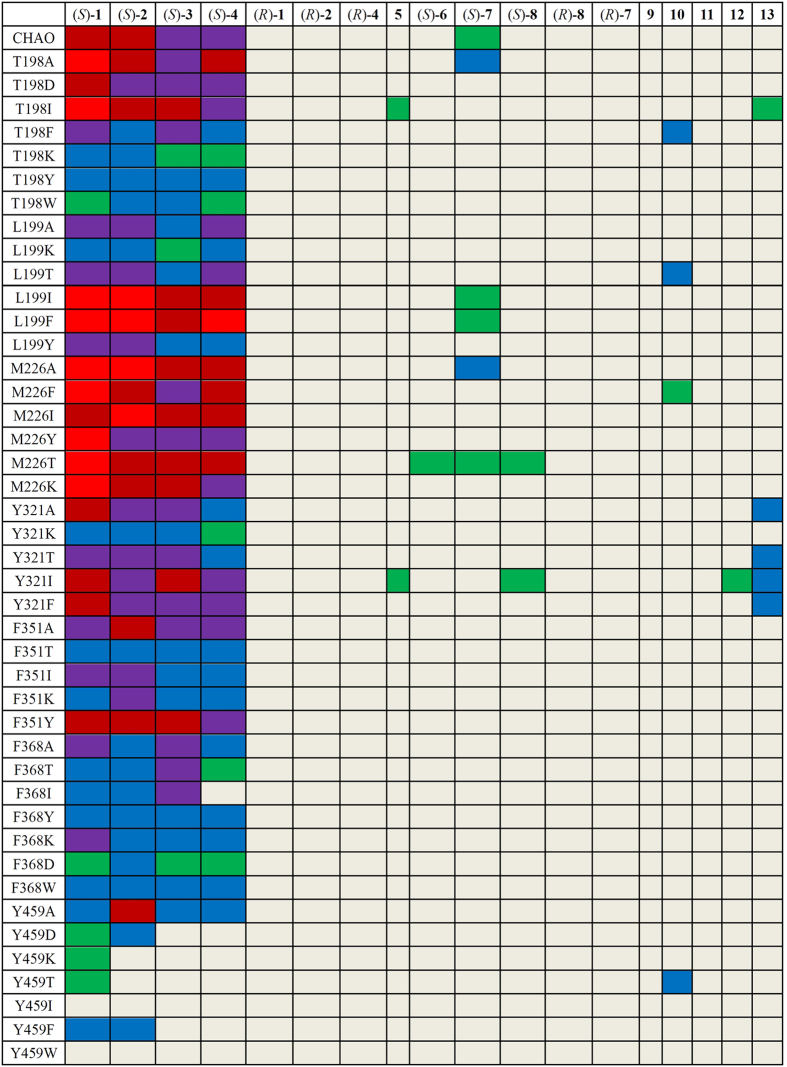
Activity assay results of CHAO and mutants against various amines. The activities of various mutants are relative to that of wt CHAO toward (*S*)-1 set as 100% (3.5U/mg). 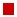 relative activity >100; 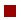 100> relative activity >50; 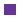 50> relative activity >10; 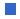 10> relative activity >1; 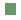 1> relative activity > 0.1; 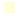 trace activity or no activity (relative activity <0.1).

Forty-one of the 51 mutants were produced as soluble proteins in *E. coli* at 25 °C, but the other mutants (L199D, L199W, M226D, M226W, Y321D, Y321W, F351D, F351W, Y459D and Y459W) were found in inclusion bodies. The trials to solubilize and refolding of the inclusion body proteins were not successful, so these mutants were not investigated further. Equal concentration of the soluble wt CHAO and 41 mutant proteins were used for activity measurements toward the selected group of substrates. These results are shown in Fig. 3 and supplementary Table S2. In general, the mutants showed a higher activity toward (*S*)-enantiomers than the (*R*)-counterparts of primary amines. Similar to wt CHAO, the mutants showed higher activity toward primary amines than the secondary ones. However, some obvious differences were observed. For example, almost all mutants of F368 and Y459 exhibited a much lower activity (46-0.1% activity relative to wt CHAO) toward (*S*)-primary amines with only exception of Y459A toward substrate (*S*)-**2**. In contrast, mutation at other selected sites generated two mutants (T198A and M226A) with higher activity than wt CHAO for most (*S*)-enantiomers of both primary amines (1.04–1.44 fold) and secondary amines (2.62–4.25 fold). Five mutants (T198I, L199I, L199F, M226I and M226T) were up to 2.1-fold more active toward the primary amines compared to the wt enzyme. The other six mutants (T198F, L199T, Y321A, Y321T, Y321I and Y321F) showed greater than 1% relative activity toward the secondary amines, which were not the active substrate for the wt enzyme.

Notably, some mutants showed an apparent activity toward bulky primary amines. For example, both mutants T198I and Y321I showed an obvious activity toward (4-chlorophenyl) (phenyl) methanamine (**5**), toward which wt CHAO was not active. Active variants were also obtained for the following secondary amines: mutant M226T for (*S*)-*N*-methyl-1-phenylethanamine ((*S*)-**6**); T198A for (*S*)-*N*-benzyl-1-phenylethylamine ((*S*)-**7**); M226T and Y321I for (*S*)-*N*-propynyl-2, 3-dihydro-1 H-inden-1-amine ((*S*)-**8**); T198F, L199T, M226T and Y459T for **10;** Y321I for 1-methyl-1, 2, 3, 4-tetrahydroisoquinoline (**12**); and T198I, Y321A, Y321T, Y321F and Y321I for **13**.

### Construction of double mutants Y321I/M226T and T198I/Y321I and the determination of kinetic parameters

Compared to wt CHAO, which was not active toward secondary amines (*S*)-**8** and **13**, some single amino acid substitutions showed activity, e.g., Y321I and M226T for (*S*)-**8**, and T198I and Y321I for **13**. To create potentially better mutants, double mutants by combining Y321I and M226T for (*S*)-**8**, and T198I and Y321I for **13**, were constructed by site-directed mutagenesis. The kinetic parameters of the purified proteins were determined to compare the catalytic efficiency of the mutants toward substrates (*S*)-**8** and **13.** As shown in Table 1, the double mutant Y321I/M226T displayed an additive effect, showing a higher catalytic efficiency toward (*S*)-**8** (930 min^−1^M^−1^ and about 1.7 and 23 fold relative to Y321I and M226T, respectively). In contrast, mutant T198I/Y321I exhibited lower catalytic efficiency compared to Y321I (about 30% relative to Y321I).

### Deracemization of 8 and 13 using crude enzyme extract of mutants Y321I/M226T and Y321I

**Figure 4:**
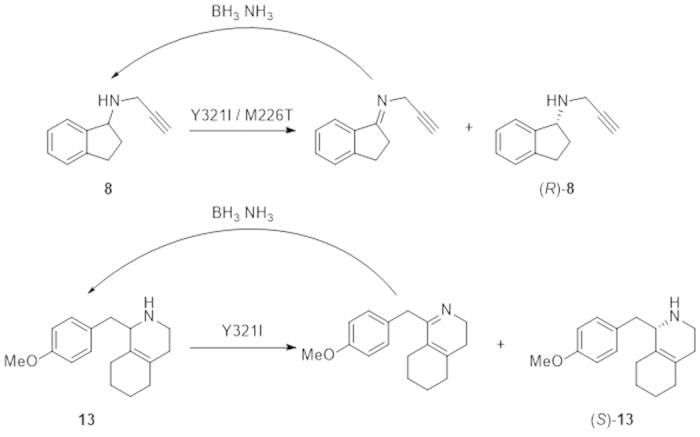
Schemes for the deracemization of N-(prop-2-yn-1-yl)-2, 3-dihydro-1H-inden-1-amine (8) or 1-(4-methoxybenzyl)-1, 2, 3, 4, 5, 6, 7, 8-octahydroisoquinoline (13) by coupling an enantioselective oxidation using an amine oxidase mutant Y321I or Y321I/M226T with a nonselective reducing agent NH_3_·BH_3_.

**Figure 5:**
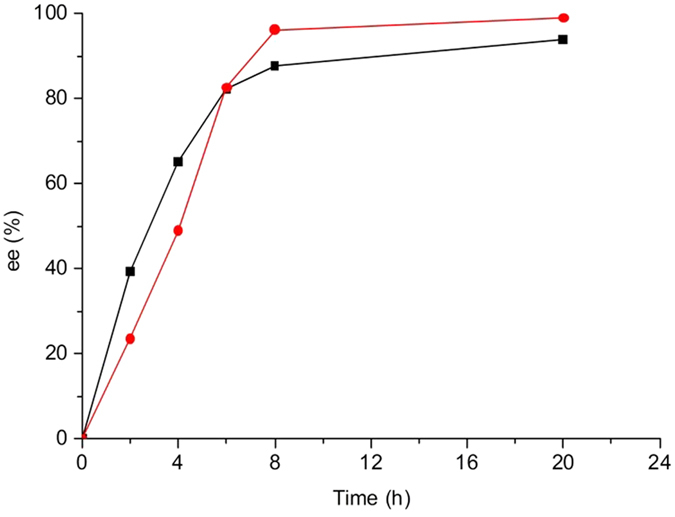
Time course of the deracemization of N-(prop-2-yn-1-yl)-2, 3-dihydro-1H-inden-1-amine (8, 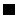) and 1-(4-methoxybenzyl)-1, 2, 3, 4, 5, 6, 7, 8-octahydroisoquinoline (13,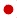).

**Figure 6:**
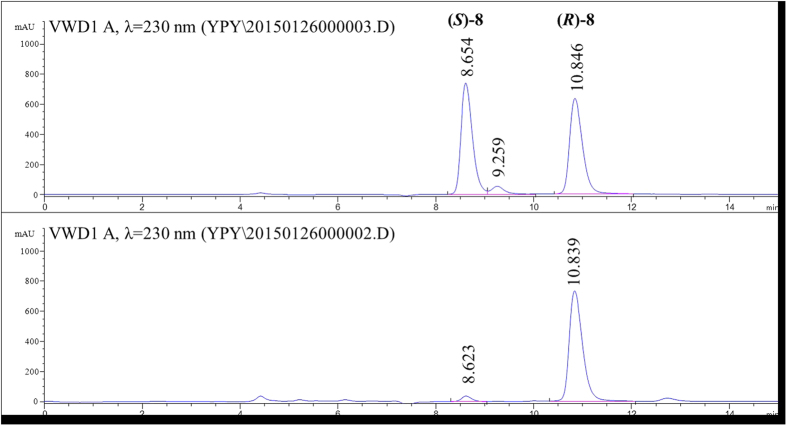
HPLC spectra of racemic 8 and the product (*R*)-*N*-(prop-2-yn-1-yl)-2, 3-dihydro-1H-inden-1-amine ((*R*)-8). The analysis was performed on a chiral OJ-H column (4.6 mm × 250 mm, DAICEL CHIRAL TECHNOLOGIES CO.LTD) with a mixture of hexane and isopropanol (0.5% ethanolamine) (9:1) as the eluent at 0.8 mL/min of flow rate and the column temperature being controlled at 30 °C.

**Figure 7:**
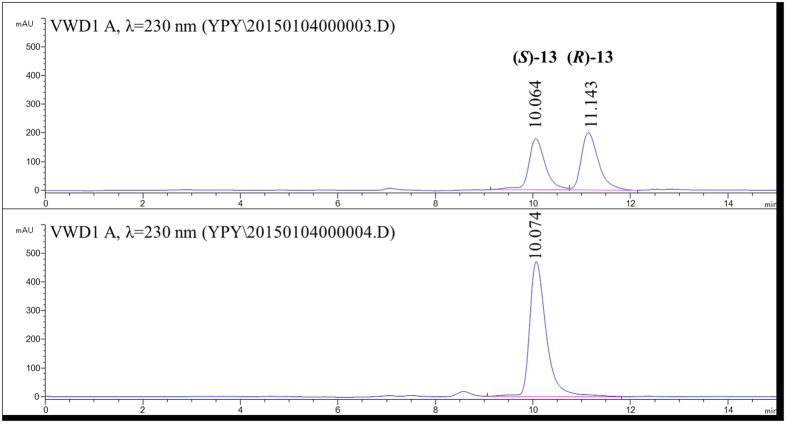
HPLC spectra of racemic 13 and the product (*S*)-1-(4-methoxybenzyl)-1, 2, 3, 4, 5, 6, 7, 8-octahydroisoquinoline ((*S*)-13). The analysis was performed on a chiral OJ-H column (4.6 mm × 250 mm, DAICEL CHIRAL TECHNOLOGIES CO.LTD) with a mixture of hexane and isopropanol (0.5% ethanolamine) (9:1) as the eluent at 0.5 mL/min of flow rate and the column temperature being controlled at 30 °C.

By virtue of the fact that mutants Y321I/M226T and Y321I displayed higher catalytic efficiency toward (*S*)-**8** and **13**, respectively, they were tested for the deracemization of **8** and **13**. Crude enzyme extracts of mutants Y321I/M226T and Y321I were coupled with the non-selective reducing agent NH_3_·BH_3_ to achieve the deracemization of **8** and **13** (Fig. 4). The reactions were fast and linear in the first eight hours, resulting in up to 88% and 96% ee for **8** and **13**, respectively. The deracemization process plateaued in the next 12 h, with slight increase in ee to 93% and 99% for **8** and **13**, respectively (Fig. 5). As a result, deracemization of **8** gave a 51% isolated yield and 93% ee of (*R*)-**8** in 20 h (Fig. 6). In the same timeframe (*S*)-**13** was prepared with 78% isolated yield and 99% ee (Fig. 7). In either case, the wt CHAO was ineffective for the said deracemization.

## Discussion

In the solved structure of CHAO, the side chains of Y321, F368 and Y459 form a cage-like structure in the substrate binding pocket, and amino acid side chains of T198, L199, M226, and F351 separate the substrate-binding cavity from a second cavity that is located closer to the protein surface^24^. In theory, these residues would affect the substrate binding profile. Indeed, previous results indicated that the amine acid residues T198, L199, M226 and Y459 exerted some effect on the enzyme’s substrate capability^15^,^16^. In those studies, five single-amino acid substitution mutants were tested with various substrates to examine the possible effects of the mutations on substrate binding and catalytic activity^15^. The mutant libraries were screened against a single substrate to identify active mutants for the deracemization of that particular chemical^16^. Herein, we created a library of diverse mutants and assayed toward a group of structurally diverse substrates to further expand the enzyme’s substrate capability and identify active mutants for deracemization of some interesting amines.

All the mutants exhibited higher activity toward (*S*)-enantiomer than the (*R*)-counterpart of primary amines, suggesting the retention of high stereoselectivity of the mutant enzymes. Most mutants of F368 and Y459 showed an obviously lower or no activity toward almost all of the tested substrates. This is consistent with the observation for the corresponding amino acid residues Y398 and Y435 of human MAO-B, which were found in the substrate binding site on the *re* face of the covalent flavin ring. Y435 in MAO-B was mutated to the amino acids F, H, L, or W, and all the mutant proteins showed an obviously decreased catalytic efficiency than the wt MAO-B^25^. The side chains of Y321, F368 and Y459 of CHAO form a cage-like structure, and previous studies have indicated that the functions of the “aromatic cage” in MAO-B include recognition of the substrate amine group and increasing the nucleophilicity of the substrate amine moiety^25^,^26^. It is possible that mutagenesis of F368 and Y459 may break the cage-like structure and result in a decrease of activity. It has been previously reported that the side chains of residues T198, L199, M226, and F351 separate the entrance cavity and the substrate-binding cavity, and play a “gate-keeping” role in CHAO^24^. For some mutants (T198A, M226A, M226I, M226T), substitutions of the original amino acids by smaller ones are envisioned to enlarge the “gate”, thus providing a reasonable basis for an enhanced activity relative to the wt CHAO. In other mutants, T198I, L199I and L199F that also displayed increased activity, we propose that those mutations might change the conformation of the “gate” between the two cavities, thus the substrate and product could diffuse more easily in and out of the substrate-binding pocket and the entrance cavity. However, without the crystal structures of these mutant proteins, it is difficult to give definitive explanations for these results.

The kinetic parameters of the mutant Y321I and Y321I/M226T proteins showed a high catalytic efficiency toward 1-(4-methoxybenzyl)-1, 2, 3, 4, 5, 6, 7, 8-octahydroisoquinoline (**13**) and (*S*)-*N*-(prop-2-yn-1-yl)-2, 3-dihydro-1 H-inden-1-amine [(*S*)-**8**], respectively. Since (*R*)-**8** is an irreversible inhibitor of monoamine oxidase for Alzheimer disease^27^, and (*S*)-**13** is a key intermediate for the synthesis of the antitussive agent dextromethorphan^28^, several methods have been reported for the synthesis of (*R*)-**8**^29^,^30^,^31^ and (*S*)-**13**^28^,^32^,^33^,^34^. Most methods are based on classical kinetic resolution with chiral acids^31^,^32^ or focused on the asymmetric synthesis of (*R*)-2, 3-dihydro-1-indanamine^29^,^30^. Kitamura *et al.* reported a stereoselective synthesis of (*S*)-**13** with 97% ee and 98% yield catalyzed by a chiral phosphine-Ru complex under high hydrogen pressure of 100 atm^34^. Broger *et al.* found that amidophosphine-phosphinites-Ir complex catalyzed asymmetric hydrogenation of the corresponding iminium salt to (*S*)-**13** in up to 86% ee^33^. Our present study is the first report that optically active (*R*)-**8** and (*S*)-**13** can be synthesized with high ee value and comparable isolated yield by an amine oxidase-catalyzed deracemization, thus providing a new biocatalytic approach to access these chiral amines and demonstrating the application potential of the evolved CHAO mutants in the synthesis of pharmaceutical molecules with chiral secondary amine moiety.

## Materials and Methods

### Materials

1-(4-methoxybenzyl)-1, 2, 3, 4, 5, 6, 7, 8-octahydroisoquinoline (**13**) was prepared as described in the literature (supplementary Figs S1 and S2)^35^. Other reagents were purchased from Sigma-Aldrich, Alfar Aesar (USA), J&K scientific Ltd (Beijing, China), and AccelaChembio Inc., and were used without further purification.

### Construction of the mutant library and double mutants Y321I/M226T and T198I/Y321I by site-specific mutagenesis

Choice of the mutation sites (T198, L199, M226, Y321, F351, F368 and Y459) was guided by the solved structure of CHAO^24^. Site-directed mutagenesis of CHAO-encoding gene in *Escherichia coli* recombinant plasmid pSD80 vector (Tac promoter)^24^ was carried out by PCR-based Quick-change method^36^. Each selected site was mutated to amino acid residues of different property (A, I, F, W, T, Y, D and K). Both of the double mutants Y321I/M226T and T198I/Y321I were constructed using Y321I plasmid as template by PCR-based Quick-change method. The mutagenic primers are shown in supplementary Table S3 and the plasmids were transformed into *E. coli* Top10. In each case, the sequence of the mutated gene was verified by DNA sequencing (BGI Company, Beijing, China).

### Expression of wt CHAO and mutants

*E. coli* recombinant cells were cultivated in 5 ml LB medium containing ampicillin (100 μg/ml) overnight at 37 °C. Then 1 mL culture was inoculated into 50 mL of LB medium containing ampicillin (100 μg/ml) and grown at 37 °C. At an OD_600_ of 0.8, gene expression was induced by the addition of IPTG (final concentration 0.5 mmol/L), and then allowed to grow for an additional 10 h at 25 °C. The cells were harvested by centrifugation at 10,000 × g and 4 °C for 15 min, then the bacterial pellet was washed twice with phosphate buffer (50 mM, pH 6.5), and resuspended in a phosphate buffer (50 mM, pH 6.5). The recombinant cells were lysed by sonication and the supernatant was collected by centrifugation at 10,000 × *g* at 4 °C for 30 min. Then the collected supernatant protein was analyzed by SDS-PAGE (supplementary Fig. S3) and protein concentration was measured by Bradford method^37^. Based on above results, the CHAO and mutants were diluted to same concentration for activity detection.

### Activity Assay

Enzyme activity was determined by a modified procedure of Braun *et al.*^38^. The formation of a dye (ε = 29.4 × 10^3^ mol^–1^ L cm^−1^) by the action of horseradish peroxidase with liberated hydrogen peroxide from the reaction of the amine and amino oxidase, 4-aminoantipyrine, and 2, 4, 6-tribromo-3-hydroxybenzoicacid was monitored at 510 nm using a SPECTRAMAX M2e (MD, USA) at 30 °C. The standard assay mixture (0.2 mL total volume) contained 184 μL of 50 mmol/L phosphate buffer (pH 6.5), 2 μL of a 2, 4, 6-tribromo-3-hydroxybenzoic acid stock solution [20 mg/mL in dimethyl sulfoxide (DMSO)], 2 μL of 4-aminoantipyrine stock solution (15 mg/mL in H_2_O), 2 μL of an amine stock solution (1 mol/L in DMSO), and 10 U of horseradish peroxidase. The reaction (0.2 mL) was started by the addition of 10 μL of an appropriate amount of enzyme at 30 °C. For each experiment, enzymatic assays were performed in triplicate with cell-free extract of *E. coli* Top10 and *E. coli* Top10 recombinant cells of wt CHAO under same culture condition as control experiments. One enzyme unit (U) was defined as the amount of enzyme that produced 1 μmol of hydrogen peroxide per min. The activity of CHAO for (*S*)-1-phenylethanamine (3.5 U/mg) was defined as 100%.

### Purification of CHAO variants and the determination of kinetic parameters

The recombinant CHAO variants (Y321I, M226T, T198I, Y321I/M226T and T198I/Y321I) were purified by ion interaction chromatography on an AKTA purifier 10 system with DEAE FF crude column (GE, USA), as reported in our previous study^15^. The purified enzymes were analyzed using SDS-PAGE (supplementary Fig. S4). The kinetic parameters were obtained by measuring the initial velocities of the enzymatic reaction and curve-fitting according to the Michaelis-Menten equation. The activity assay was performed in a mixture containing a varying concentration of (*S*)-**8** (0.1-10 mM) or **13** (0.1-10 mM). All experiments were conducted in triplicate.

### General procedure for deracemization of 8 and 13 using crude enzyme extract of mutant Y321I/M226T and Y321I

The plasmids of mutants Y321I or Y321I/M226T were transformed into *E. coli* BL21(DE3). The cells were cultured and the crude enzyme extracts were prepared as described above and used for biotransformation. The deracemization of **8** or **13** was carried out as follows: **8** (118 mg, 0.75 mmol) or **13** (129 mg, 0.5 mmol) dissolved in DMSO (1 mL), 50 mL crude enzyme extract of mutant Y321I/M226T for **8** or Y321I for **13** (10 g wet cells) in phosphate buffer (50 mM, pH = 6.5) and borane–ammonia complex (124 mg, 4 mmol) were mixed. The mixture was shaken at 200 rpm at 25 °C on an orbital shaker. After 20 h of reaction, the reaction was quenched with 6 M HCl to pH 2 and the pH of the mixture was then adjusted to 10 with 5 M NaOH. The aqueous layer was extracted two times with 25 mL of ethyl acetate and the phase separation was facilitated by centrifugation (5500 × *g*, 15 min). The combined organic extract was dried over anhydrous sodium sulphate, filtered, and evaporated in vacuum. The product was purified by preparative thin layer chromatography.

Deracemization of **8** gave 60 mg product (51% isolated yield, ee = 93%). Optical rotation for (*R*)-**8**: [α]^25^_D_ = 17.2 (c = 1.7, chloroform). Literature values for (*R*)-**8**: [α]^25^_D_ = 18.8 (c = 1.7, chloroform)^39^. HPLC analysis was performed on an Agilent 1200 Series using Chiracel OJ-H column (4.6 mm × 250 mm, DAICEL CHIRAL TECHNOLOGIES CO.LTD).A mixture of hexane and isopropanol (0.5% ethanolamine) (9:1) was used as eluent in 0.8 mL/min of flow rate and the column temperature was controlled at 30 °C. The retention times for (*S*)-**8** and (*R*)-**8** were 8.61 and 10.84 min, respectively. ^1^H NMR (400 MHz, CDCl_3_): δ 7.31–7.37 (m, 1 H), 7.15–7.24 (m, 3 H), 4.41 (t, *J* = 6.1 Hz, 1 H), 3.52 (t, *J* = 2.3 Hz, 2 H), 2.98–3.09 (m, 1 H), 2.78–2.88 (m, 1 H), 2.34–2.45 (m, 1 H), 2.25 (t, *J* = 2.3 Hz, 1 H), 1.81–1.91 (m, 1 H), 1.71 (s, 1 H). ^13^C NMR (100 MHz, CDCl_3_): δ 144.51, 143.84, 127.66, 126.28, 124.89, 124.22, 82.50, 71.42, 61.90, 36.17, 33.34, 30.48 (supplementary Figs S5 and S6).

Deracemization of **13** gave 100 mg product (78% isolated yield, ee > 99%), which was identified as (*S*)-**13** by comparison of its optical rotation with the literature^40^. Observed values for (*S*)-**13**: [α]^20^_D_ = −130 (c = 1.0, methanol). Literature values for (*R*)-**13**: [α]^20^_D_ = 139 (c = 1.0, methanol). HPLC analysis was performed on an Agilent 1200 Series using Chiracel OJ-H column (4.6 mm × 250 mm, DAICEL CHIRAL TECHNOLOGIES CO.LTD). A mixture of hexane and isopropanol (0.5% ethanolamine) (9:1) was used as eluent in 0.5 mL/min of flow rate and the column temperature was controlled at 30 °C. The retention times for (*S*)-**13** and (*R*)-**13** were 10.06 and 11.14 min, respectively. ^1^H NMR (400 MHz, CDCl_3_): δ 7.21 (d, *J* = 8.6 Hz, 2 H), 6.85 (d, *J* = 8.6 Hz, 2 H), 3.79 (s, 3 H), 3.52 (br. s., 1 H), 3.08 (dd, *J* = 14.1, 4.4 Hz, 1 H), 2.93–3.02 (m, 1 H), 2.87 (dt, *J* = 12.0, 5.9 Hz, 1 H), 2.78 (dd, *J* = 14.1, 8.4 Hz, 1 H), 1.98–2.15 (m, 3 H), 1.94 (br. s., 2 H), 1.82 (m, 1 H), 1.63–1.77 (m, 2 H), 1.55 (m, 2 H). ^13^C NMR (100 MHz, CDCl_3_): δ 158.40, 130.46, 129.88, 128.83, 127.45, 114.06, 57.56, 55.23, 39.57, 37.05, 30.21, 28.72, 27.07, 22.85, 22.53 (supplementary Figs S7 and S8).

## Additional Information

**How to cite this article**: Li, G. *et al.* New recombinant cyclohexylamine oxidase variants for deracemization of secondary amines by orthogonally assaying designed mutants with structurally diverse substrates. *Sci. Rep.* **6**, 24973; doi: 10.1038/srep24973 (2016).

## Supplementary Material

Supplementary Information

## Figures and Tables

**Table 1:** Kinetic parameters of cyclohexylamine oxidase variants.

Substrate	Variant	*K*m (mM)	*k*_cat_ (min^−1^)	*k*_cat_/*K*m (min^−1^ M^−1^)
(*S*)-8	Y321I	2.22 ± 0.32	1.21 ± 0.06	540
	M226T	5.03 ± 0.46	0.20 ± 0.06	40
	Y321IM226T	0.43 ± 0.05	0.41 ± 0.02	930
13	T198I	n.d.	n.d.	n.d.
	Y321I	0.89 ± 0.09	0.35 ± 0.01	390
	T198IY321I	4.93 ± 0.75	0.68 ± 0.06	140

n.d: not determined due to negligible activity.
