# Supplementary material for: New recombinant cyclohexylamine oxidase variants for deracemization of secondary amines by orthogonally assaying designed mutants with structurally diverse substrates
Source: Sci Rep. 2016 May 3;6:24973. doi: 10.1038/srep24973 (PMC4853734; doi:10.1038/srep24973)
Supplement: Supplementary Information [file srep24973-s1.pdf]

New recombinant cyclohexylamine oxidase variants for deracemization of secondary amines by orthogonally assaying designed mutants with structurally diverse substrates

Guangyue Li[†,1], Peiyuan Yao[†,1], Peiqian Cong[1], Jie Ren[1], Lei Wang[1], JinhuiFeng[1], Peter C.K. Lau[*,1,2], Qiaqing Wu[*,1], and Dunming Zhu[*,1]

[1] National Engineering Laboratory for Industrial Enzymes and Tianjin Engineering Center for Biocatalytic Technology, Tianjin Institute of Industrial Biotechnology, Chinese Academy of Sciences, 32 Xi Qi Dao, Tianjin Airport Economic Area, Tianjin 300308, PR China

[2] Departments of Chemistry and Microbiology & Immunology, McGill University, Montreal, Quebec, H3A2B4, Canada

[†] The authors contributed equally to this work

Contents

Table S1 List of mutants 2

Table S2 Substrate specificity of the mutant enzymes 2

Table S3 Primers used for mutation....................8

Fig. S1 ^{1}H-NMR spectra of 1-(4-methoxybenzyl)-1, 2, 3, 4, 5, 6, 7, 8-octahydroisoquinoline (**13**)....................11

Fig. S2 ^{13}C-NMR spectra of 1-(4-methoxybenzyl)-1, 2, 3, 4, 5, 6, 7, 8-octahydroisoquinoline (**13**)....................12

Fig. S3 SDS-PAGE analysis of the cell-free extracts of CHAO and mutants....................12

Fig. S4 SDS-PAGE analysis of the purified CHAO and mutants 13

Fig. S5 ^{1}H-NMR spectra of (*S*)-*N*-(prop-2-yn-1-yl)-2, 3-dihydro-1H-inden-1-amine ((*R*)-8) 13

Fig. S6 ^{13}C-NMR spectra of (*S*)-*N*-(prop-2-yn-1-yl)-2, 3-dihydro-1H-inden-1-amine ((*R*)-8) 14

Fig. S7 ^{1}H-NMR spectra of (*S*)-1-(4-methoxybenzyl)-1, 2, 3, 4, 5, 6, 7, 8-octahydroisoquinoline ((*S*)-13)....................14

Fig. S8 ^{13}C-NMR spectra of (*S*)-1-(4-methoxybenzyl)-1, 2, 3, 4, 5, 6, 7, 8-octahydroisoquinoline ((*S*)-13)....................15

Table S1 List of mutants

T198	L199	M226	Y321	F351	F368	Y459
T198A	L199A	M226A	Y321A	F351A	F368A	Y459A
T198D	L199D	M226D	Y321D	F351D	F368D	Y459D
T198K	L199K	M226K	Y321K	F351K	F368K	Y459K
T198F	L199T	M226T	Y321T	F351T	F368T	Y459T
T198I	L199I	M226I	Y321I	F351Y	F368Y	Y459I
T198W	L199F	M226F	Y321F	F351I	F368I	Y459F
T198Y	L199Y	M226Y	Y321W	F351W	F368W	Y459W
	L199W	M226W				

Table S2 Substrate specificity of the mutant enzymes

A. Substrate specificity of CHAO mutants at 198. The activity of CHAO for (*S*)-1-phenylethanamine was defined as 100% (3.5U/mg) and the result showed in sequence of CHAO/198A/198D/198I/198F/198K/198Y/198W.

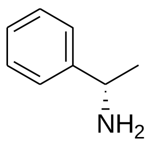	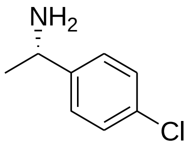	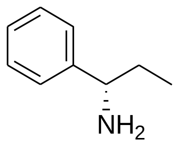	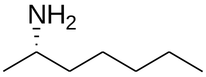	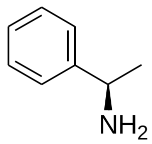
**(*S*)-1**	**(*S*)-2**	**(*S*)-3**	**(*S*)-4**	**(*R*)-1**
100±6.91/113.26±4.06/77.23±4.76/134.46±5.23/10.11±0.13/1.12±0.02/2.70±0.02/0.79±0.01	87.31±5.30/90.87±5.21/20.95±1.53/93.12±0.97/9.11±0.62/2.44±1.29/2.20±0.19/1.65±0.02	46.74±3.30/36±2.78/12.25±0.62/64.66±4.50/14.21±0.42/0.79±0.05/2.22±0.14/2.48±0.55	47.95±2.24/52.71±1.23/20.95±3.98/43.76±1.78/3.96±0.21/0.26±0.05/1.05±0.24/0.21±0.05	T/T/T/T/T/T/T/T
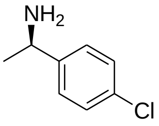	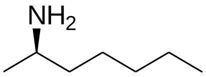	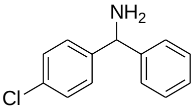	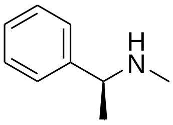	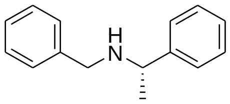
**(*R*)-2**	**(*R*)-4**	**5**	**(*S*)-6**	**(*S*)-7**
T/T/T/T/T/T/T/T	T/T/T/T/T/T/T/T	T/T/T/0.72±0.05/T/T/T/T	N/N/N/N/N/N/N/N	0.36±0.05/1.53±0.05/T/T/T/T/T/T
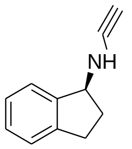	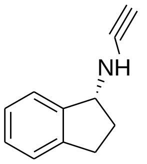	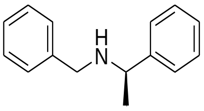	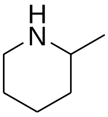	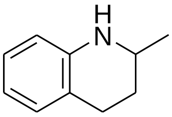
**(*S*)-8**	**(*R*)-8**	**(*R*)-7**	**9**	**10**
N/N/N/N/N/N/N/N	N/N/N/N/N/N/N/N	N/N/N/N/N/N/N/N	N/N/N/N/N/N/N/N	T/T/T/T/1.45±0.21/T/T/T

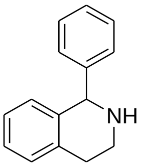	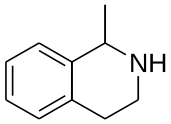	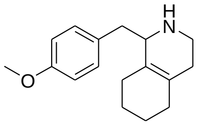
**11**	**12**	**13**
T/T/T/T/T/T/T	T/T/T/T/T/T/T	T/T/T/0.49±0.08/T/T/T

T, trace activity (below 0.1%).

N，the activity was not detected.

B. Substrate specificity of CHAO mutants at 199. The activity of CHAO for (*S*)-1-phenylethanamine was defined as 100% (3.5U/mg) and the result showed in sequence of CHAO/199A/199K/199T/199I/199F/199Y.

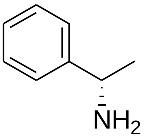	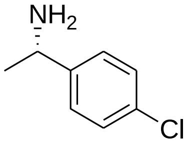	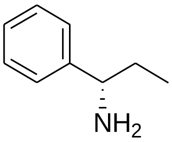	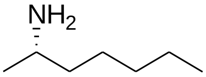	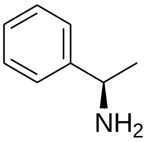
**(*S*)-1**	**(*S*)-2**	**(*S*)-3**	**(*S*)-4**	**(*R*)-1**
100±5.35/40.91±3.12/1.1±0.042/28.26±1.23/134.22±6.74/178.33±8.34/21.41±2.01	83.32±7.04/13.61±1.25/3.12±0.15/44.72±5.34/112.2±11.24/145.42±11.54/27.91±4.01	42.55±1.57/5.91±0.14/0.63±0.02/6.25±0.71/84.91±7.64/71.92±3.54/4.62±0.08	49.82±4.38/25.23±3.19/1.13±0.04/22.35±4.01/69.33±5.43/104.83±6.75/9.13±0.43	T/T/T/T/T/T/T
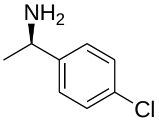	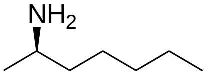	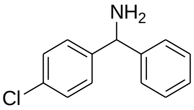	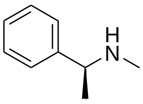	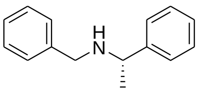
**(*R*)-2**	**(*R*)-4**	**5**	**(*S*)-6**	**(*S*)-7**
T/T/T/T/T/T/T	T/T/T/T/T/T/T	T/T/T/T/T/T/T	N/N/N/N/N/N/N	0.32±0.01/N/N/N/0.93±0.04/0.44±0.02/N
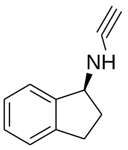	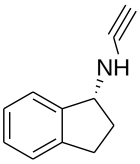	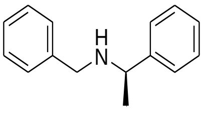	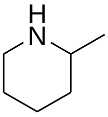	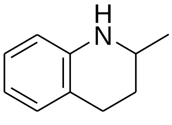
**(*S*)-8**	**(*R*)-8**	**(*R*)-7**	**9**	**10**
N/N/N/N/N/N/N	N/N/N/N/N/N/N	N/N/N/N/N/N/N	N/N/N/N/N/N/N	T/T/T/2.23±0.08/T/T/T
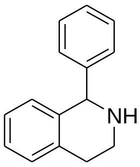	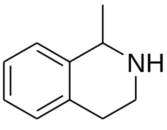	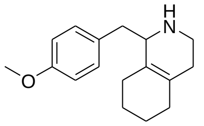		
**11**	**12**	**13**		
T/T/T/T/T/T/T	T/T/T/T/T/T/T	T/T/T/T/T/T/T		

T, trace activity (below 0.1%).

N，the activity was not detected.

C. Substrate specificity of CHAO mutants at 226. The activity of CHAO for (*S*)-1-phenylethanamine was defined as 100% (3.5U/mg) and the result showed in sequence of CHAO/226A/226F/226I/226Y/226T/226K.

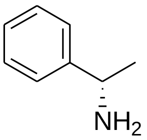	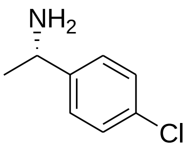	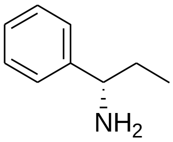	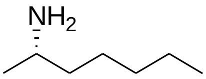	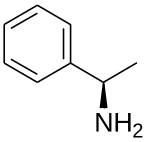
**(*S*)-1**	**(*S*)-2**	**(*S*)-3**	**(*S*)-4**	**(*R*)-1**
100±3.82/116.97±1.71/114.14±1.28/116.10±3.09/84.78±3.81/103.41±11.26/108.94±1.65	84.46±0.52/104.11±6.95/55.59±3.14/100.93±5.97/38.67±3.38/90.88±1.69/76.20±2.56	49.28±3.22/69.22±7.22/46.46±1.78/51.54±2.19/30.44±3.58/97.85±2.42/52.64±2.63	42.17±1.77/60.59±2.55/64.14±3.37/69.26±2.75/34.66±5.21/75.03±7.66/18.26±2.28	T/T/T/T/T/T/T
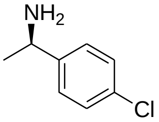	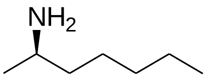	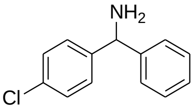	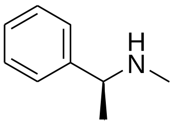	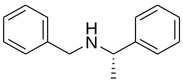
**(*R*)-2**	**(*R*)-4**	**5**	**(*S*)-6**	**(*S*)-7**
T/T/T/T/T/T/T	T/T/T/T/T/T/T	T/T/T/T/T/T/T	N/N/N/N/N/0.51±0.06/N	0.53±0.07/1.39±0.12/T/T/T/0.93±0.07/T
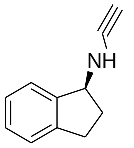	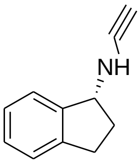	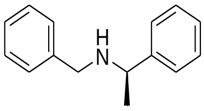	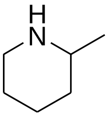	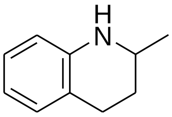
**(*S*)-8**	**(*R*)-8**	**(*R*)-7**	**9**	**10**
N/N/N/N/N/0.48±0.02/N	N/N/N/N/N/N/N	N/N/N/N/N/N/N	N/N/N/N/N/N/N	T/T/0.94±0.03/T/T/T/T
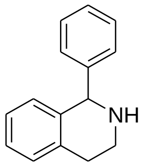	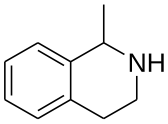	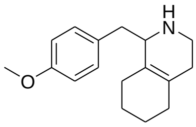		
**11**	**12**	**13**		
T/T/T/T/T/T/T	T/T/T/T/T/T/T	T/T/T/T/T/T/T		

T, trace activity (below 0.1%).

N，the activity was not detected.

D. Substrate specificity of CHAO mutants at Y321. The activity of CHAO for (*S*)-1-phenylethanamine was defined as 100% (3.5U/mg) and the result showed in sequence of CHAO/321A/321K/321T/321I/321F.

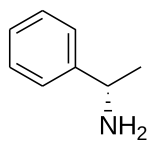	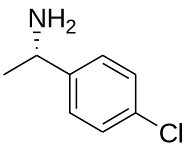	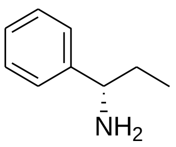	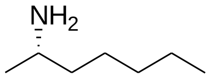	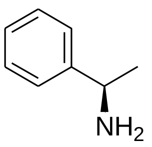
**(*S*)-1**	**(*S*)-2**	**(*S*)-3**	**(*S*)-4**	**(*R*)-1**
100±9.08/52.53±6.58/5.63±0.41/20.86±1.28/86.24±6.25/62.23±4.73	84.62±7.32/12.13±0.16/1.94±0.12/10.62±0.18/36.93±0.52/26.86±0.95	43.13±1.59/32.32±0.78/2.93±0.15/26.16±1.67/84.22±5.07/29.56±0.93	46.26±3.34/7.76±0.17/0.82±0.07/2.31±0.16/19.16±0.91/14.11±0.96	T/T/T/T/T/T
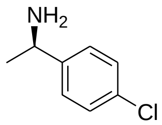	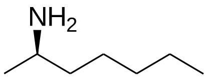	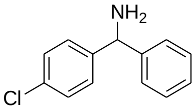	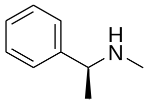	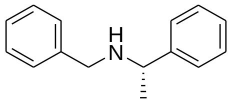
**(*R*)-2**	**(*R*)-4**	**5**	**(*S*)-6**	**(*S*)-7**
T/T/T/T/T/T	T/T/T/T/T/T	T/T/T/T/0.62±0.05/T	N/N/N/N/N/N	0.36±0.04/T/T/T/T/T
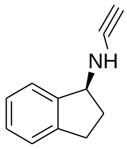	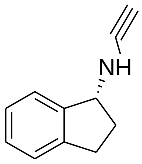	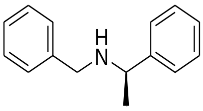	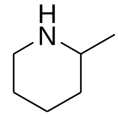	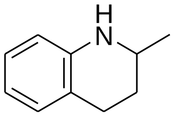
**(*S*)-8**	**(*R*)-8**	**(*R*)-7**	**9**	**10**
N/N/N/N/0.37±0.08/N	N/N/N/N/N/N	N/N/N/N/N/N	N/N/N/N/N/N	T/T/T/T/T/T
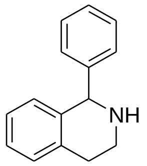	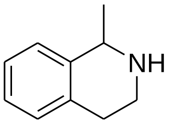	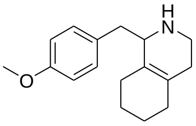		
**11**	**12**	**13**		
T/T/T/T/T/T	T/T/T/T/0.33±0.01/T	T/1.21±0.02/T/1.34±0.12/2.55±0.05/1.33±0.14		

T, trace activity (below 0.1%).

N，the activity was not detected.

E. Substrate specificity of CHAO mutants at F351. The activity of CHAO for (*S*)-1-phenylethanamine was defined as 100% (3.5U/mg) and the result showed in sequence of CHAO/351A/351T/351I/351K/351Y.

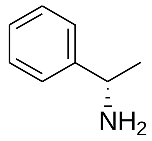	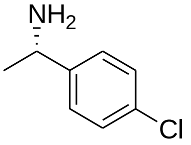	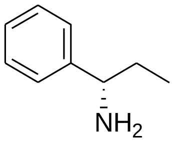	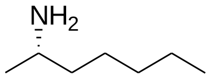	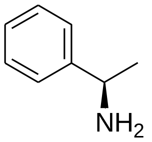

**(*S*)-1**	**(*S*)-2**	**(*S*)-3**	**(*S*)-4**	**(*R*)-1**
100±6.41/28.25±0.38/3.06±0.03/10.51±0.39/6.03±0.04/89.56±9.38	83.83±6.31/57.91±0.59/3.82±0.12/16.83±1.34/31.75±2.43/67.13±6.83	42.35±1.42/22.23±0.84/1.59±0.21/9.16±0.89/2.71±0.21/54.82±2.96	44.21±2.36/29.17±2.97/1.78±0.10/6.81±0.13/6.02±0.95/40.43±6.33	T/T/T/T/T/T
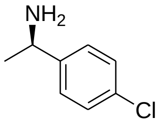	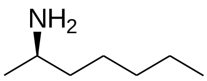	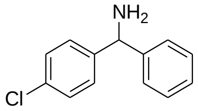	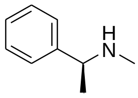	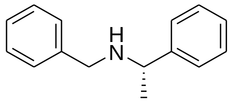
**(*R*)-2**	**(*R*)-4**	**5**	**(*S*)-6**	**(*S*)-7**
T/T/T/T/T/T	T/T/T/T/T/T	T/T/T/T/T/T	N/N/N/N/N/N	0.36±0.03/T/T/T/T/T
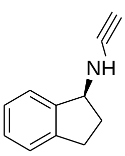	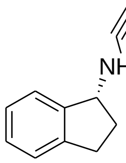	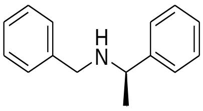	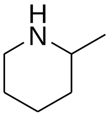	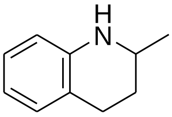
**(*S*)-8**	**(*R*)-8**	**(*R*)-7**	**9**	**10**
N/N/N/N/N/N	N/N/N/N/N/N	N/N/N/N/N/N	N/N/N/N/N/N	T/T/T/T/T/T
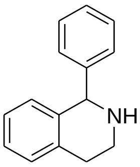	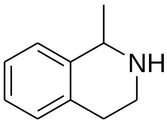	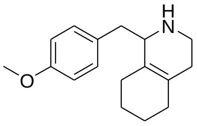		
**11**	**12**	**13**		
T/T/T/T/T/T	T/T/T/T/T/T	T/T/T/T/T/T		

T, trace activity (below 0.1%).

N，the activity was not detected.

F. Substrate specificity of CHAO mutants at Y368. The activity of CHAO for (*S*)-1-phenylethanamine was defined as 100% (3.5U/mg) and the result showed in sequence of CHAO/368A/368T/368I/368Y/368K/368D/368W.

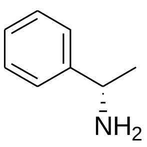	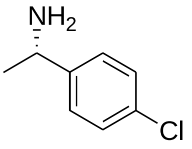	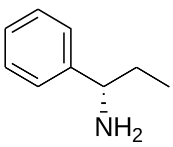	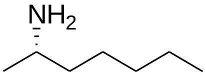	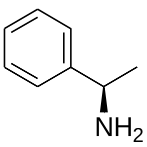
**(*S*)-1**	**(*S*)-2**	**(*S*)-3**	**(*S*)-4**	**(*R*)-1**
100±6.12/15.04±1.65/4.58±0.16/2.02±0.25/1.85±0.21/14.25±1.82/0.97±0.05/4.79±0.16	82.34±4.19/7.08±1.07/3.20±0.42/1.16±0.13/1.76±0.45/9.89±0.30/1.38±0.13/4.09±0.38	45.83±1.27/12.05±0.86/21.49±1.36/12.63±0.57/2.64±0.21/6.93±0.15/0.56±0.05/2.77±0.38	44.04±3.81/3.41±0.56/0.74±0.16/T/2.22±0.47/8.72±0.81/0.15±0.02/3.47±0.59	T/T/T/T/T/T/T/T

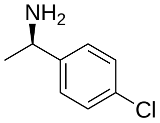	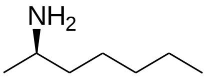	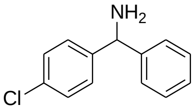	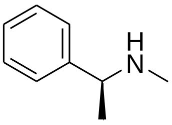	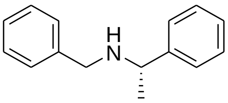
**(*R*)-2**	**(*R*)-4**	**5**	**(*S*)-6**	**(*S*)-7**
T/T/T/T/T/T/T/T	T/T/T/T/T/T/T/T	T/T/T/T/T/T/T/T	N/N/N/N/N/N/N/N	0.38 ±0.02/T/T/T/T/T/T/T
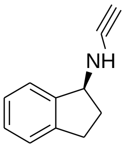	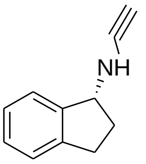	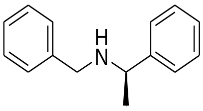	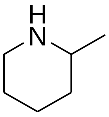	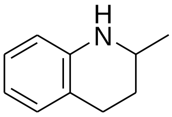
**(*S*)-8**	**(*R*)-8**	**(*R*)-7**	**9**	**10**
N/N/N/N/N/N/N/N	N/N/N/N/N/N/N/N	N/N/N/N/N/N/N/N	N/N/N/N/N/N/N/N	T/T/T/T/T/T/T/T
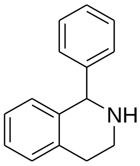	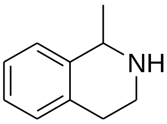	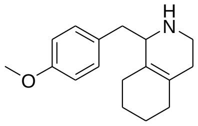		
**11**	**12**	**13**		
T/T/T/T/T/T/T/T	T/T/T/T/T/T/T/T	T/T/T/T/T/T/T/T		

T, trace activity (below 0.1%).

N，the activity was not detected.

G. Substrate specificity of CHAO mutants at Y459. The activity of CHAO for (*S*)-1-phenylethanamine was defined as 100% (3.5U/mg) and the result showed in sequence of CHAO/459A/459D/459K/459T/459I/459F/459W.

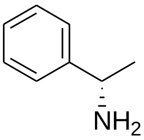	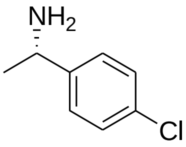	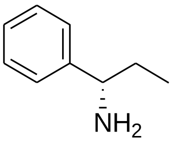	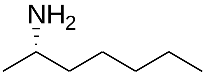	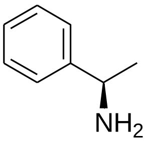
**(*S*)-1**	**(*S*)-2**	**(*S*)-3**	**(*S*)-4**	**(*R*)-1**
100 ±5.36/6.71 ±0.87/0.55 ±0.12/0.16 ±0.02/0.32 ±0.02/N/3.49 ±0.29/N	83.76 ±6.21/80.11 ±4.18/5.11 ±0.51/N/N/N/7.27 ±1.01/N	42.77 ±3.58/2.59 ±0.25/N/N/N/N/N	44.56 ±3.26/3.14 ±0.15/N/N/N/N/N	T/N/N/N/N/N/N/N
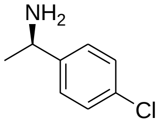	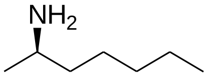	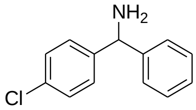	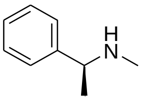	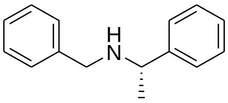
**(*R*)-2**	**(*R*)-4**	**5**	**(*S*)-6**	**(*S*)-7**
T/N/N/N/N/N/N/N	T/N/N/N/N/N/N/N	T/N/N/N/N/N/N/N	T/N/N/N/N/N/N/N	0.36 ±0.02/N/N/N/N/N/N/N

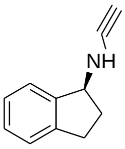	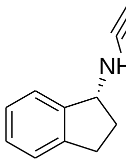	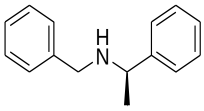	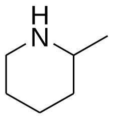	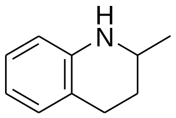
**(*S*)-8**	**(*R*)-8**	**(*R*)-7**	**9**	**10**
N/N/N/N/N/N/N/N	N/ N/N/N/N/N/N/N	N/N/N/N/N/N/N/N	N/N/N/N/N/N/N/N	T/N/N/N/3.41 ±0.21/N/N/N
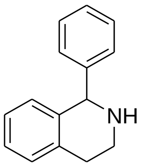	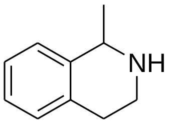	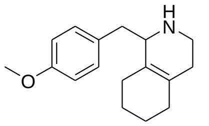		
**11**	**12**	**13**		
T/N/N/N/N/N/N/N	T/N/N/N/N/N/N/N	T/N/N/N/N/N/N/N		

T, trace activity (below 0.1%).

N，the activity was not detected.

Table S3 Primers used for mutation

Primer	sequence(5'to3')
T198F-5’	ACGGTTATCGTCAATTTCCTCCTCGGCGCCGA
T198F-3’	TCGGCGCCGAGGAGGAAATTGACGATAACCGT
T198A-5’	ACGGTTATCGTCAATGCGCTCCTCGGCGCCGA
T198A-3’	TCGGCGCCGAGGAGCGCATTGACGATAACCGT
T198W-5’	ACGGTTATCGTCAATTGGCTCCTCGGCGCCGA
T198W-3’	TCGGCGCCGAGGAGCCAATTGACGATAACCGT
T198I-5’	ACGGTTATCGTCAATATCCTCCTCGGCGCCGA
T198I-3’	TCGGCGCCGAGGAGGATATTGACGATAACCGT
T198Y-5’	ACGGTTATCGTCAATTATCTCCTCGGCGCCGA
T198Y-3’	TCGGCGCCGAGGAGATAATTGACGATAACCGT
T198K-5’	ACGGTTATCGTCAATAAGCTCCTCGGCGCCGA
T198K-3’	TCGGCGCCGAGGAGCTTATTGACGATAACCGT
T198D-5’	ACGGTTATCGTCAATGACCTCCTCGGCGCCGA
T198D-3’	TCGGCGCCGAGGAGGTCATTGACGATAACCGT
L199F-5’	GTTATCGTCAATACCTTCCTCGGCGCCGACCCCTA
L199F-3’	TAGGGGTCGGCGCCGAGGAAGGTATTGACGATAAC
L199W-5’	GTTATCGTCAATACCTGGCTCGGCGCCGACCCCTA
L199W-3’	TAGGGGTCGGCGCCGAGCCAGGTATTGACGATAAC

Primer	sequence(5'to3')
L199I-5’	GTTATCGTCAATACCATCCTCGGCGCCGACCCCTA
L199I-3’	TAGGGGTCGGCGCCGAGGATGGTATTGACGATAAC
L199Y-5’	GTTATCGTCAATACCTATCTCGGCGCCGACCCCTA
L199Y-3’	TAGGGGTCGGCGCCGAGATAGGTATTGACGATAAC
L199T-5’	GTTATCGTCAATACCACCCTCGGCGCCGACCCCTA
L199T-3’	TAGGGGTCGGCGCCGAGGGTGGTATTGACGATAAC
L199K-5’	GTTATCGTCAATACCAAGCTCGGCGCCGACCCCTA
L199K-3’	TAGGGGTCGGCGCCGAGCTTGGTATTGACGATAAC
L199D-5’	GTTATCGTCAATACCGACCTCGGCGCCGACCCCTA
L199D-3’	TAGGGGTCGGCGCCGAGGTCGGTATTGACGATAAC
L199A-5’	GTTATCGTCAATACCGCGCTCGGCGCCGACCCCTA
L199A-3’	TAGGGGTCGGCGCCGAGCGCGGTATTGACGATAAC
M226F 5’	AAGGGATACAGTCACTCTTCGGCACCCGGGACGGA
M226F 3’	TCCGTCCCGGGTGCCGAAGAGTGACTGTATCCCTT
M226W 5’	AAGGGATACAGTCACTCTGGGGCACCCGGGACGGA
M226W 3’	TCCGTCCCGGGTGCCCCAGAGTGACTGTATCCCTT
M226I 5’	AAGGGATACAGTCACTCATCGGCACCCGGGACGGA
M226I 3’	TCCGTCCCGGGTGCCGATGAGTGACTGTATCCCTT
M226Y 5’	AAGGGATACAGTCACTCTATGGCACCCGGGACGGA
M226Y 3’	TCCGTCCCGGGTGCCATAGAGTGACTGTATCCCTT
M226T 5’	AAGGGATACAGTCACTCACCGGCACCCGGGACGGA
M226T 3’	TCCGTCCCGGGTGCCGGTGAGTGACTGTATCCCTT
M226K 5’	AAGGGATACAGTCACTCAAGGGCACCCGGGACGGA
M226K 3’	TCCGTCCCGGGTGCCCTTGAGTGACTGTATCCCTT
M226D 5’	AAGGGATACAGTCACTCGACGGCACCCGGGACGGA
M226D 3’	TCCGTCCCGGGTGCCGTCGAGTGACTGTATCCCTT
M226A 5’	AAGGGATACAGTCACTCGCGGGCACCCGGGACGGA
M226A 3’	TCCGTCCCGGGTGCCCGCGAGTGACTGTATCCCTT
Y321W 5’	GCACCGATGGGCCGCTGGTACAAGGTGCAGGC
Y321W 3’	GCCTGCACCTTGTACCAGCGGCCCATCGGTGC
Y321I 5’	GCACCGATGGGCCGCATCTACAAGGTGCAGGC
Y321I 3’	GCCTGCACCTTGTAGATGCGGCCCATCGGTGC
Y321T 5’	GCACCGATGGGCCGCACCTACAAGGTGCAGGC
Y321T 3’	GCCTGCACCTTGTAGGTGCGGCCCATCGGTGC

Primer	sequence(5'to3')
Y321K 5’	GCACCGATGGGCCGCAAGTACAAGGTGCAGGC
Y321K 3’	GCCTGCACCTTGTACTTGCGGCCCATCGGTGC
Y321D 5’	GCACCGATGGGCCGCGACTACAAGGTGCAGGC
Y321D 3’	GCCTGCACCTTGTAGTCGCGGCCCATCGGTGC
Y321F 5’	GCACCGATGGGCCGCTTCTACAAGGTGCAGGC
Y321F 3’	GCCTGCACCTTGTAGAAGCGGCCCATCGGTGC
Y321A 5’	GCACCGATGGGCCGCGCGTACAAGGTGCAGGC
Y321A 3’	GCCTGCACCTTGTACGCGCGGCCCATCGGTGC
F351A 5’	GAAGACGTCGGAGTCGCGCTCCTCGACGGCACG
F351A 3’	CGTGCCGTCGAGGAGCGCGACTCCGACGTCTTC
F351W 5’	GAAGACGTCGGAGTCTGGCTCCTCGACGGCACG
F351W 3’	CGTGCCGTCGAGGAGCCAGACTCCGACGTCTTC
F351I 5’	GAAGACGTCGGAGTCATCCTCCTCGACGGCACG
F351I 3’	CGTGCCGTCGAGGAGGATGACTCCGACGTCTTC
F351Y 5’	GAAGACGTCGGAGTCTATCTCCTCGACGGCACG
F351Y 3’	CGTGCCGTCGAGGAGATAGACTCCGACGTCTTC
F351T 5’	GAAGACGTCGGAGTCACCCTCCTCGACGGCACG
F351T 3’	CGTGCCGTCGAGGAGGGTGACTCCGACGTCTTC
F351K 5’	GAAGACGTCGGAGTCAAGCTCCTCGACGGCACG
F351K 3’	CGTGCCGTCGAGGAGCTTGACTCCGACGTCTTC
F351D 5’	GAAGACGTCGGAGTCGACCTCCTCGACGGCACG
F351D 3’	CGTGCCGTCGAGGAGGTCGACTCCGACGTCTTC
F368A 5’	AGCGACCCTAATTGGGGCGATCGGAGGGTCAAATT
F368A 3’	AATTTGACCCTCCGATCGCCCCAATTAGGGTCGCT
F368W 5’	AGCGACCCTAATTGGGTGGATCGGAGGGTCAAATT
F368W 3’	AATTTGACCCTCCGATCCACCCAATTAGGGTCGCT
F368I 5’	AGCGACCCTAATTGGGATCATCGGAGGGTCAAATT
F368I 3’	AATTTGACCCTCCGATGATCCCAATTAGGGTCGCT
F368Y 5’	AGCGACCCTAATTGGGTATATCGGAGGGTCAAATT
F368Y 3’	AATTTGACCCTCCGATATACCCAATTAGGGTCGCT
F368T 5’	AGCGACCCTAATTGGGACCATCGGAGGGTCAAATT
F368T 3’	AATTTGACCCTCCGATGGTCCCAATTAGGGTCGCT
F368K 5’	AGCGACCCTAATTGGGAAGATCGGAGGGTCAAATT
F368K 3’	AATTTGACCCTCCGATCTTCCCAATTAGGGTCGCT
F368D 5’	AGCGACCCTAATTGGGGACATCGGAGGGTCAAATT
F368D 3’	AATTTGACCCTCCGATGTCCCCAATTAGGGTCGCT
Y459W 5’	TCCAATGGTCCGGCTGGATGGAAGGTGGCGT
Y459W 3’	ACGCCACCTTCCATCCAGCCGGACCATTGGA
Y459I 5’	TCCAATGGTCCGGCATCATGGAAGGTGGCGT
Y459I 3’	ACGCCACCTTCCATGATGCCGGACCATTGGA

Primer	sequence(5'to3')
Y459T 5’	TCCAATGGTCCGGCACCATGGAAGGTGGCGT
Y459T 3’	ACGCCACCTTCCATGGTGCCGGACCATTGGA
Y459K 5’	TCCAATGGTCCGGCAAGATGGAAGGTGGCGT
Y459K 3’	ACGCCACCTTCCATCTTGCCGGACCATTGGA
Y459D 5’	TCCAATGGTCCGGCGACATGGAAGGTGGCGT
Y459D 3’	ACGCCACCTTCCATGTCGCCGGACCATTGGA
Y459F 5’	TCCAATGGTCCGGCTTCATGGAAGGTGGCGT
Y459F 3’	ACGCCACCTTCCATGAAGCCGGACCATTGGA
Y459A 5’	TCCAATGGTCCGGCGCGATGGAAGGTGGCGT
Y459A 3’	ACGCCACCTTCCATCGCGCCGGACCATTGGA

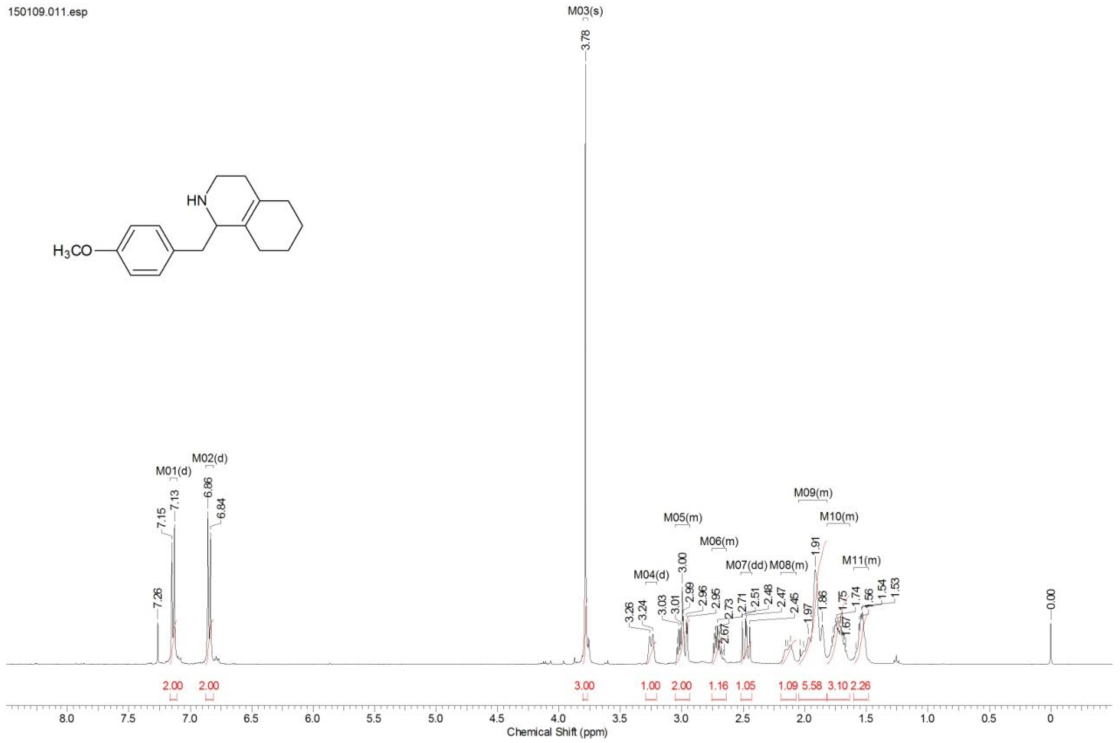

Fig. S1 ^{1}H-NMR spectra of 1-(4-methoxybenzyl)-1, 2, 3, 4, 5, 6, 7, 8-octahydroisoquinoline (**13**)

^{1}H NMR (600 MHz, DMSO-d6)δ: 7.13 (d, J=8.44 Hz, 2 H), 6.84 (d, J=8.44 Hz, 2 H), 3.72 (s, 3 H), 3.13 (d, J=9.54 Hz, 1 H), 2.89 (dt, J=11.83, 5.64 Hz, 1 H), 2.85 (dd, J=13.57, 2.93 Hz, 1 H), 2.57 - 2.63 (m, 1 H), 2.38 (dd, J=13.39, 10.09 Hz, 1 H), 2.08 (d, J=14.67 Hz, 1 H), 1.74 - 1.92 (m, 5 H), 1.59 - 1.73 (m, 2 H), 1.40 - 1.51 (m, 2 H).

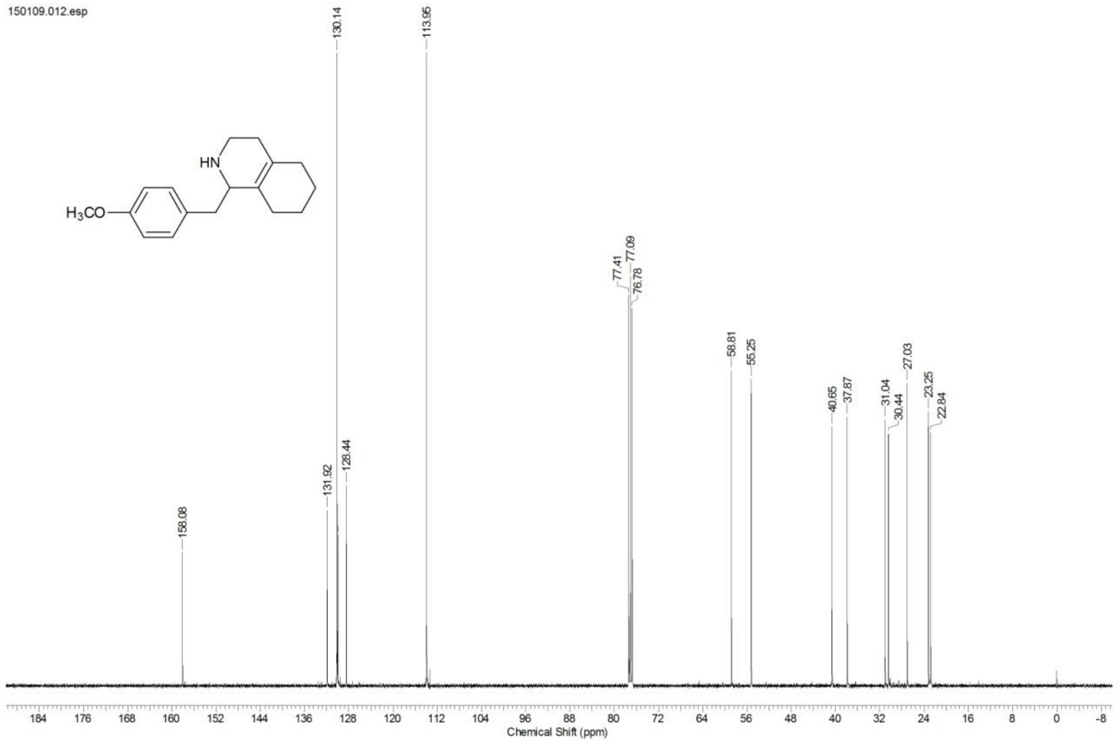

Fig. S2 ^{13}C-NMR spectra of 1-(4-methoxybenzyl)-1, 2, 3, 4, 5, 6, 7, 8-octahydroisoquinoline (**13**)

^{13}C NMR (150 MHz, DMSO-d6) δ:157.38, 131.92, 130.21, 130.01, 127.44, 113.44, 58.15, 54.83, 37.37, 30.50, 29.82, 26.43, 22.74, 22.39.

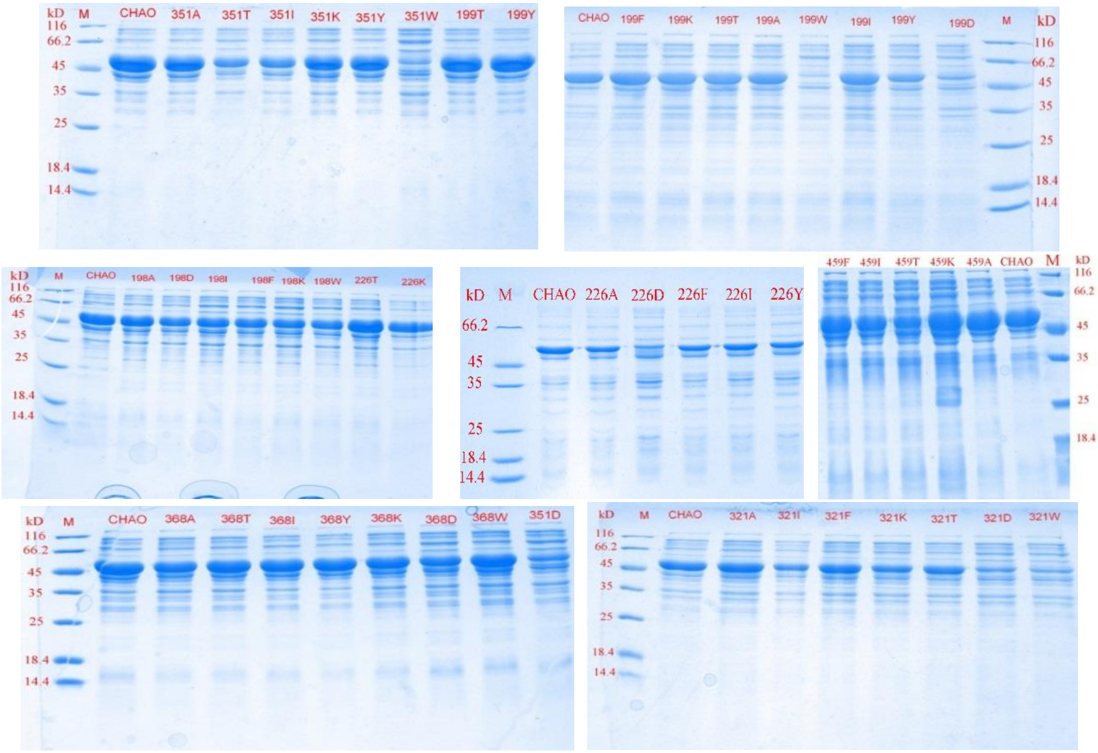

Fig. S3 SDS-PAGE analysis of the cell-free extracts of CHAO and mutants

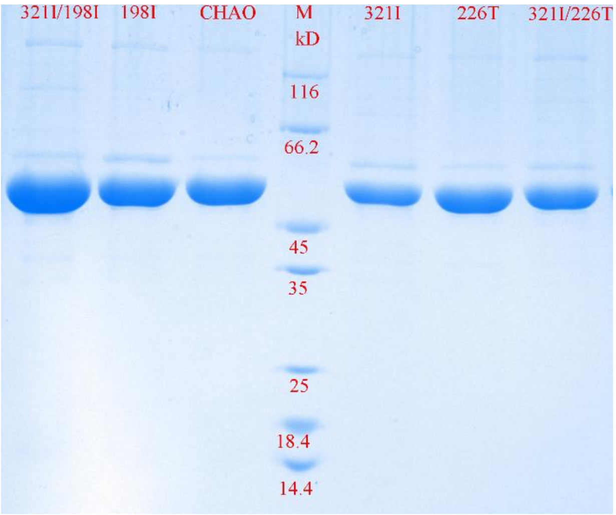

Fig. S4 SDS-PAGE analysis of the purified CHAO and mutants

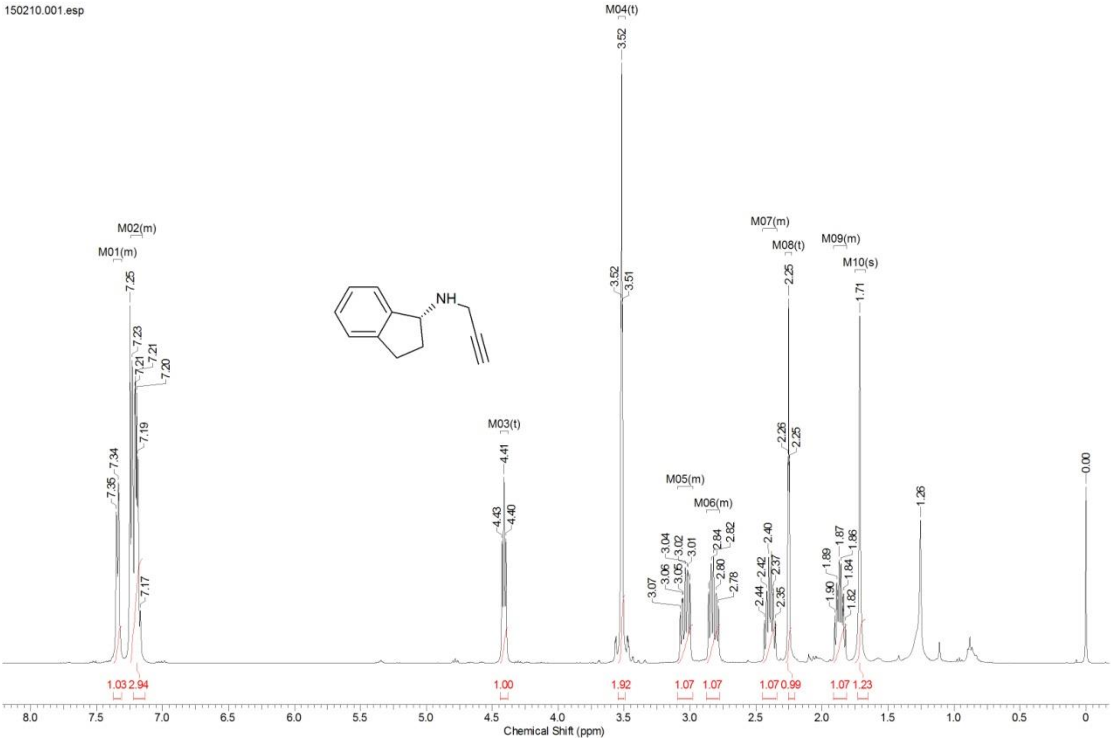

Fig. S5 ^{1}H-NMR spectra of (*S*)-*N*-(prop-2-yn-1-yl)-2, 3-dihydro-1H-inden-1-amine ((*R*)-**8**)

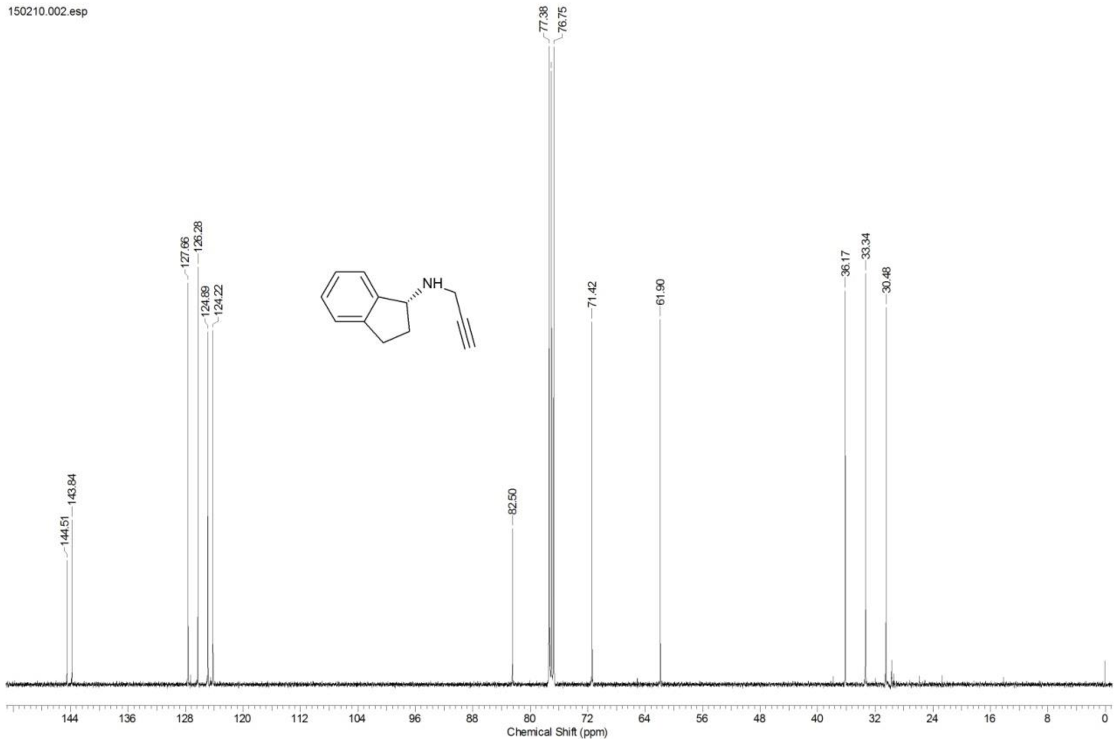

Fig. S6 ^{13}C-NMR spectra of (*S*)-*N*-(prop-2-yn-1-yl)-2, 3-dihydro-1H-inden-1-amine ((*R*)-**8**)

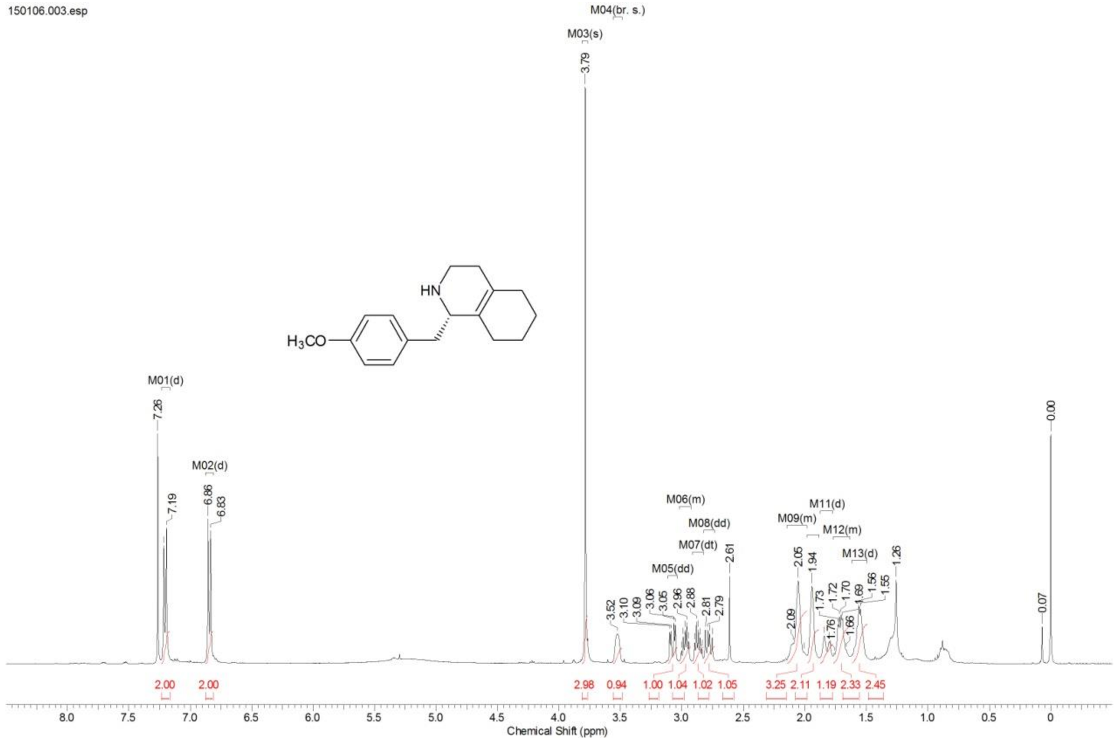

Fig. S7 ^{1}H-NMR spectra of (*S*)-1-(4-methoxybenzyl)-1, 2, 3, 4, 5, 6, 7, 8-octahydroisoquinoline ((*S*)-**13**)

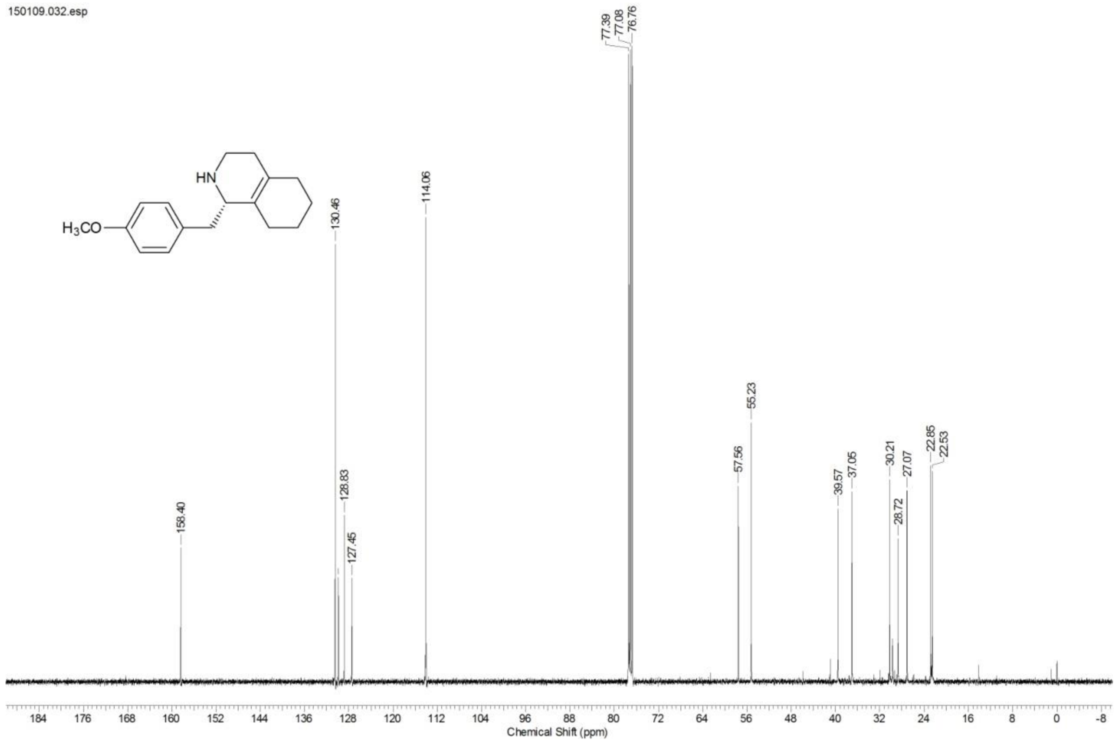

Fig. S8 ^{13}C-NMR spectra of (*S*)-1-(4-methoxybenzyl)-1, 2, 3, 4, 5, 6, 7, 8-octahydroisoquinoline ((*S*)-**13**)
